# Second-Order Nonlinearity in Triangular Lattice Perforated Gold Film due to Surface Plasmas Resonance

**DOI:** 10.1155/2014/284929

**Published:** 2014-02-16

**Authors:** Renlong Zhou, Xiaoshuang Chen, Yingyi Xiao, Bingju Zhou, Lingxi Wu, Xiaojuan Liu, Yongyi Gao, Jie Zhan

**Affiliations:** ^1^School of Physics and Electronic Science, Hunan University of Science and Technology, Xiangtan 411201, China; ^2^National Laboratory for Infrared Physics, Shanghai Institute of Technical Physics, Chinese Academy of Sciences, Shanghai 200083, China

## Abstract

We have studied the excitation second-order nonlinearity through a triangular lattice perforated gold film instead of square lattice in many papers. Under the excitation of surface plasmas resonance effect, the second order nonlinearity exists in the noncentrosymmetric split-ring resonators arrays. Reflection of fundamental frequency wave through a triangular lattice perforated gold film is obtained. We also described the second harmonic conversion efficiencies in the second order nonlinear optical process with the spectra. Moreover, the electric field distributions of fundamental frequency above the gold film region are calculated. The light propagation through the holes results in the enhancement of the second order nonlinearity including second harmonic generation as well as the sum (difference) frequency generation.

## 1. Introduction

Nonlinear optical responses have been subject of intensive studies due to the development of nanofabrication techniques [1–6]. Second harmonic generation (SHG) is a powerful tool for probing physical and structure properties of the interface and surface of materials [7–12]. The SHG in square lattice with different structures, such as double-hole arrays or semiconductor nanoparticles arrays, has also been well studied [12, 13]. The underlying physical mechanism of SHG in metal nanoparticles has been explored numerically and experimentally [14–18]. The plasmonic oscillations of the conduction electrons inside the metal can induce the localized surface plasmon (SP) resonances. These localized surface plasmon (SP) resonances play important role in the process of nonlinear optical responses. The SHG from different SP resonance configurations such as split-ring resonators [17, 18], sharp metal tips [19, 20], metallodielectric multilayer structures [21], imperfect spheres [22, 23], and L-shaped and T-shaped nanoparticles [9, 24–26] has been investigated. The theory of second harmonic generation (SHG) in three-dimensional structures consisting of arbitrary distributions of metallic spheres made of centrosymmetric materials is developed by means of multiple scattering of electromagnetic multipole fields [27]. Other theoretical methods with various kinds of approaches for the SHG have been developed by using the finite difference time domain (FDTD) method [13].

In this work, we present the surface plasmas excitation of the SHG in three-dimensional triangular lattice structures consisting of noncentrosymmetric split-ring resonators. We obtained the reflection spectra of fundamental frequency wave through a triangular lattice structures consisting of split-ring resonators. We also described the SH conversion efficiencies in the second-order nonlinear optical process with the spectra of second harmonic generation field. We find that the SH conversion efficiencies of SHG signal are in the range of about 10^−12^–10^−14^. The electric field distributions of fundamental frequency and second harmonic are also calculated by FDTD simulation. We have investigated the second-harmonic generation (SHG), sum frequency generation (SFG), and difference frequency generation (DFG). The enhanced SP resonance causes the increase of local second-order nonlinearity.

## 2. Theory and Method

Second-order nonlinearity of split-ring resonators nanostructures has been studied with the classical theoretical method in [13]. We consider the three-dimensional triangular lattice structures consisting of gold split-ring resonators.

The permittivity *ε*_*r*_(*ω*) of the gold split-ring resonators has the form

(1) $$\begin{array}{c} \varepsilon_{r}\left(\omega \right)=1-\frac{\omega_{p}^{2}}{\omega \left(\omega +i\gamma \right)}. \end{array}$$

The *γ* is collision frequency, and $w_{p}=\sqrt{e^{2}n_{0}/m_{e}\varepsilon_{0}}$ is the plasma frequency of gold. The plasma frequency and collision frequency are taken as *w*_*p*_ = 1.367 × 10^16^ s^−1^ and = 6.478 × 10^13^ s^−1^, respectively.

There are two computational loops for the calculations of the fundamental and second harmonic fields in the FDTD program. The FDTD method to calculate the first-order field at the fundamental frequency has been discussed in [13]. The FDTD approach is also applied for the numerical calculation of the second-order equations. If we consider the second-order nonlinearity of the gold, the electric and magnetic field can be obtained as follows:

(2) ∂B(2)∂t=−∇×E(2),  ∂E(2)∂t=c2∇×B(2)−1ε0j(2),j(2)=−iωp(2)=−iωε0(εr−1)E(2)+S(2),∂j(2)∂t=−γj(2)+e2n0meE(2)+S(2),S(2)=∑k∂∂rk(j(1)j(1)ken0) −eme[ε0(∇·E(1))E(1)+j(1)×B(1)].

Here, *k* represents the *x*,  *y*, and *z* coordinates. *J* ^(1)^ and *J* ^(2)^ represent the current density vectors of fundamental and harmonic waves, respectively. *E* ^(1)^ and *E* ^(2)^,  *B* ^(1)^, and *B* ^(2)^ are the electric field and magnetic flux intensity vectors of fundamental and harmonic waves, respectively. *n*_0_ is the ion density, *m*_*e*_ is the electron mass, and *S* ^(2)^ is the nonlinear source of the plasma for second-order nonlinearity, respectively.

**Figure 1:**
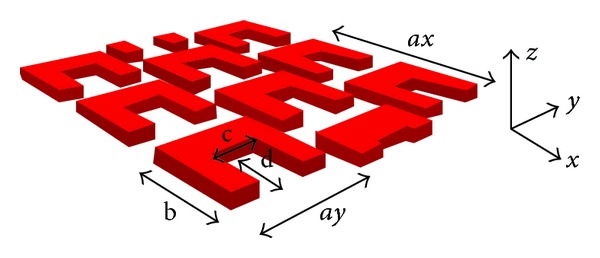
The structure of a triangular lattice structures consisting of gold split-ring resonators with the thickness *h* = 30.5 nm and the lattice periodic *a* = *ax* = *ay* = 305 nm. There is the unit cell shape with *b* = 219 nm, *c* = 131 nm and *d* = 97 nm. The input light wave is polarized along the *y* direction and propagates along the *z* direction.

The structure of a triangular lattice perforated gold film is shown in Figure 1. The perfectly matched absorbing boundary conditions are employed at the bottom and top of the computational space along the *z* direction, and the periodic boundary conditions are used on the boundaries of *x* and *y* directions. The incident wave is polarized along the *y* direction and propagates along the *z*-axis. The triangular lattice structure consists of split-ring resonators with the thickness *h* = 30.5 nm and the lattice periodic *a* = *ax* = *ay* = 305 nm. The unit cell shape of split-ring resonator has *b* = 219 nm, *c* = 131 nm, and *d* = 97 nm. The input light wave is polarized along the *y* direction and propagates along the *z* direction.

## 3. Results and Discussions

**Figure 2:**
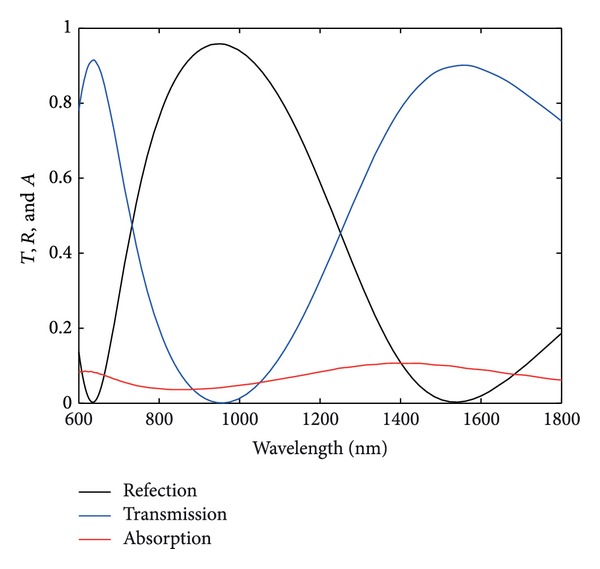
Reflection (transmission and absorption) spectra of fundamental frequency wave through a triangular lattice perforated gold film.

First, the normalized reflection (transmission and absorption) spectra of fundamental frequency wave through a triangular lattice perforated gold film are investigated here, and the calculation results are shown in Figure 2. There are two different SP resonance modes at the wavelengths 636 nm and 1548 nm for reflection spectra. These localized SP resonances at the wavelengths 636 nm and 1548 nm play important role in the process of nonlinear optical responses. The nonlinear optical responses from different SP resonance in split-ring resonators can result as different second-order nonlinearity.

To obtain the SHG with a triangular lattice perforated gold film patterns, the input light wave *E*_*y*_^(1)^ is polarized along the y direction with wavelengths *λ*_1_ or *λ*_2_:

(3) $$\begin{array}{c} E_{y}^{\left(1\right)}=E_{0}\sin\left(\frac{2\pi ct}{\lambda_{1,2}}\right), \end{array}$$

where *E*_0_ is amplitude. We consider the wavelengths *λ*_1_ = 636 nm or *λ*_2_ = 1548 nm in (3) in order to satisfy the transmission of the fundamental frequency waves, respectively.

**Figure 3:**
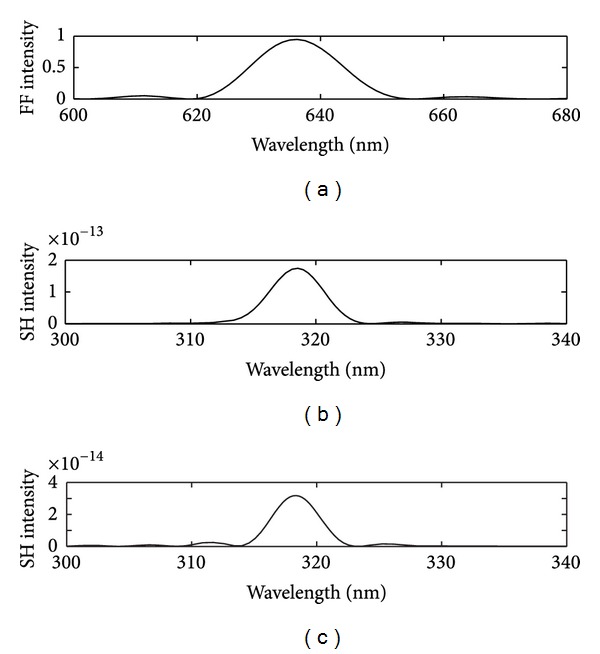
(a) The FTS for *E*_*y*_^(1)^ component of fundamental frequency field at the wavelength 636 nm. The FTS for (b) *E*_*x*_^(2)^ and (c) *E*_*y*_^(2)^ component of second-harmonic generation at the wavelength 318 nm.

**Figure 4:**
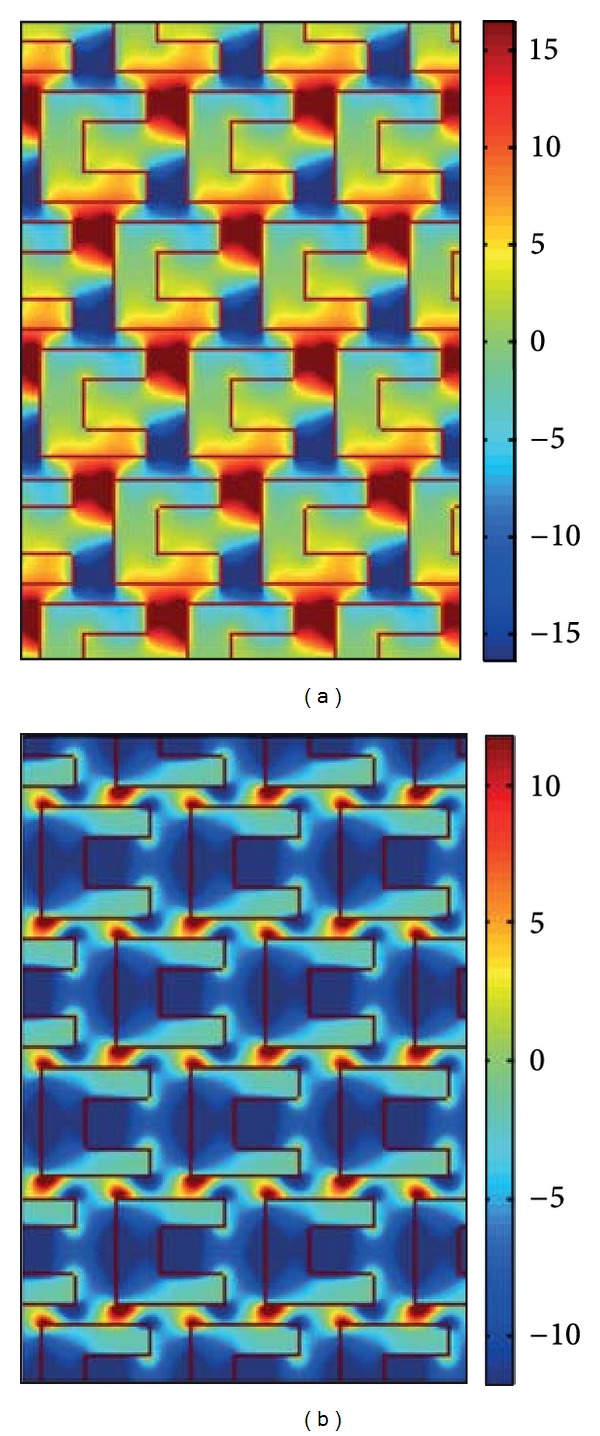
The electric field (a) *E*_*x*_^(1)^and (b) *E*_*y*_^(1)^ distribution of fundamental frequency above the gold film region at wavelength 636 nm, respectively.

When the continuous wave *E*_*y*_^(1)^ at wavelength *λ*_1_ = 636 nm is incident through the triangular lattice perforated gold film, one can see Fourier transform spectrum (FTS) of fundamental frequency wave at the wavelength 636 nm in Figure 3(a). The FTS of the *E*_*x*_^(2)^ and the *E*_*y*_^(2)^ component of SHG at the wavelength 318 nm in Figures 3(b)-3(c), respectively, are also shown. To describe the SH conversion efficiencies in the second-order nonlinear optical process, the normalized SH intensity is defined as follows:

(4) $$\begin{array}{c} \eta ={\left|\frac{E^{\left(2\right)}\left(2\omega_{0}\right)}{E^{\left(1\right)}\left(\omega_{0}\right)}\right|}^{2}, \end{array}$$

where *ω*_0_ is the freqency of the incident FF wave. The *x*-polarized SH conversion efficiencies are about 10^−13^ while the *y*-polarized SH conversion efficiencies are about 10^−14^ for the fundamental frequency wave at the wavelength 636 nm as shown in Figures 3(b)-3(c). The electric field distributions of *E*_*x*_^(1)^ and *E*_*y*_^(1)^ for fundamental frequency field above the gold film region at wavelengths 636 nm are also shown in Figures 4(a)-4(b), respectively.

**Figure 5:**
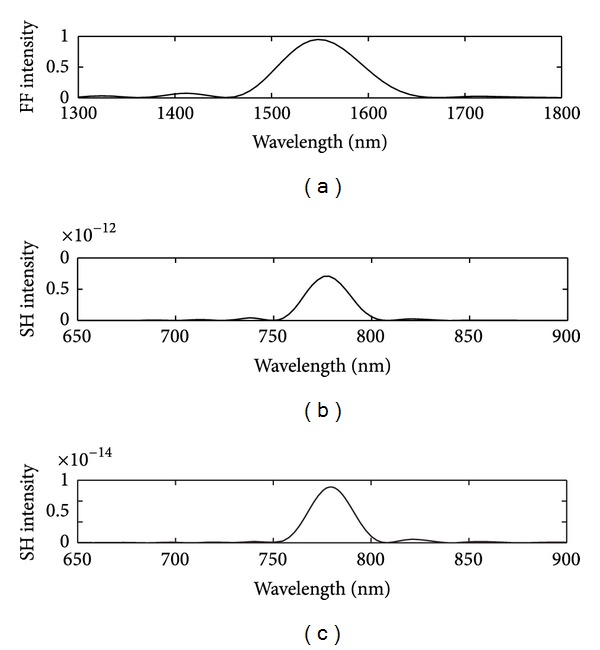
(a) The FTS for *E*_*y*_^(1)^ component of fundamental frequency wave at the wavelength 1548 nm. The FTS for (b) *E*_*x*_^(2)^ and (c) *E*_*y*_^(2)^ component of second-harmonic generation at the wavelength 774 nm.

**Figure 6:**
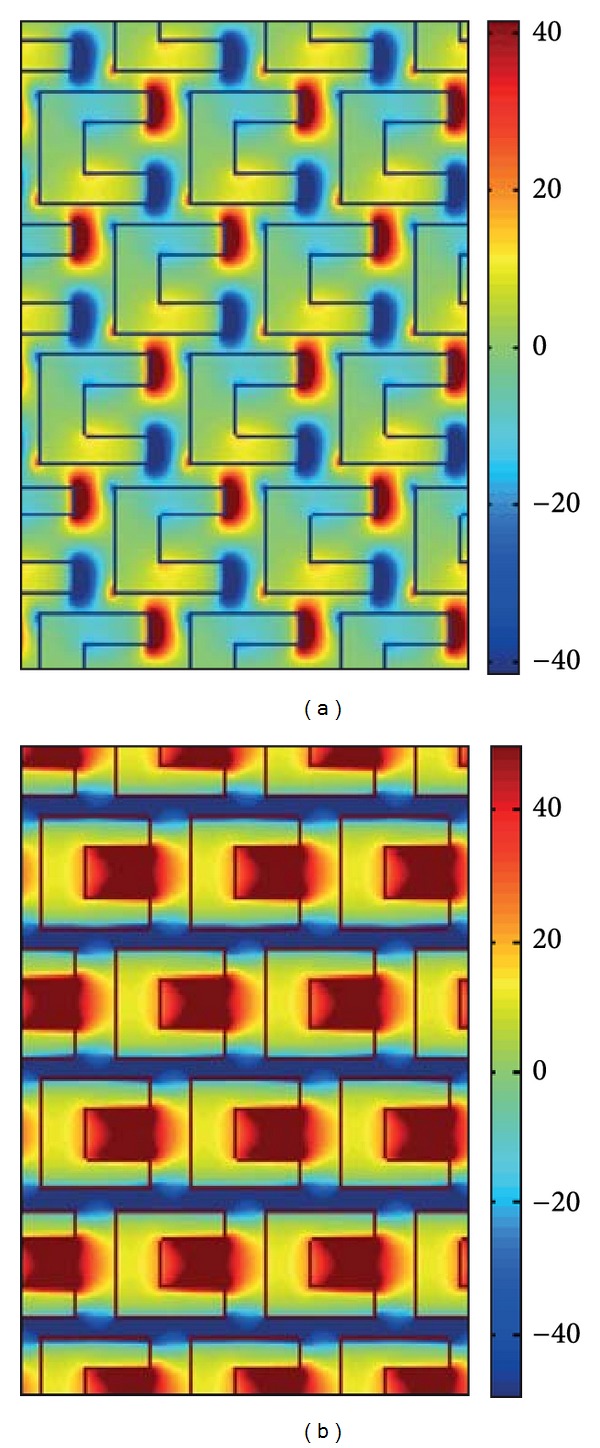
The electric field (a) *E*_*x*_^(1)^and (b) *E*_*y*_^(1)^ distribution of fundamental frequency above the gold film region at wavelength 1548 nm, respectively.

When the continuous wave *E*_*y*_^(1)^ at wavelengths *λ*_1_ = 1548 nm is incident through the triangular lattice perforated gold film, one can see FTS of fundamental frequency wave at the wavelength 1548 nm in Figure 5(a). The FTS of the *E*_*x*_^(2)^and the *E*_*y*_^(2)^ component of SHG at the wavelength 774 nm in Figures 5(b)-5(c), respectively, are also shown. The *x*-polarized SH conversion efficiencies are about 10^−12^ while the *y*-polarized SH conversion efficiencies are about 10^−14^ for the fundamental frequency wave at the wavelength 1548 nm as shown in Figures 5(b)-5(c). The electric field distribution of *E*_*x*_ and *E*_*y*_ for fundamental frequency field above the gold film region at wavelengths 1548 nm is also shown in Figures 6(a)-6(b), respectively.

To obtain the second-order nonlinearity including second-harmonic generation as well as the sum (difference) frequency generation of the triangular lattice gold film with split-ring resonators patterns, the input light wave *E*_*y*_^(1)^ is polarized along the *y* direction with two different wavelengths *λ*_1_ and *λ*_2_

(5) $$\begin{array}{c} E_{y}^{\left(1\right)}=E_{0}\sin\left(\frac{2\pi ct}{\lambda_{1}}\right)+E_{0}\sin\left(\frac{2\pi ct}{\lambda_{2}}\right), \end{array}$$

where *E*_0_ is amplitude. We consider two wavelengths *λ*_1_ = 636 nm and *λ*_2_ = 1548 nm in (5).

**Figure 7:**
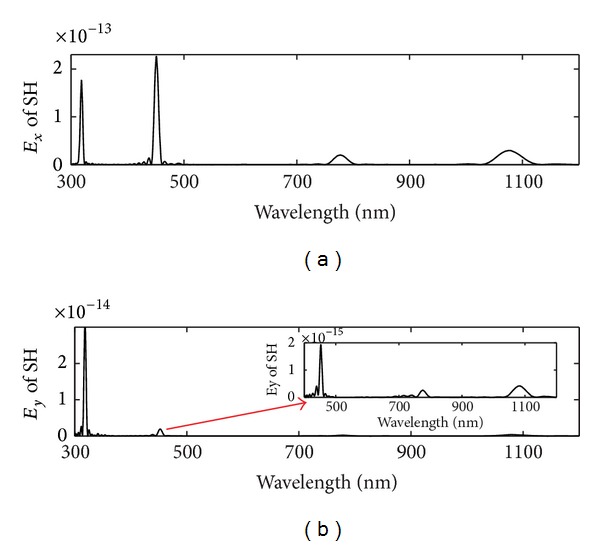
The FTS of second-order nonlinearity signals at the wavelength 318 nm, 451 nm, 774 nm, and 1080 nm for the two continuous wave *E*_*y*_^(1)^ incidence at the wavelengths *λ*_1_ = 636 nm and *λ*_2_ = 1548 nm, respectively.

When the continuous wave *E*_*y*_^(1)^ with two different wavelengths 636 nm and 1548 nm is incident through the triangular lattice perforated gold film, it is found that there are four peaks for second-order nonlinearity at the wavelengths 318 nm, 451 nm, 774 nm, and 1080 nm in Figure 7, respectively. The second-order nonlinearity modes at wavelengths 318 nm and 774 nm are obtained from the SP modes of two incident continuous waves with wavelengths 636 nm and 1548 nm due to the SHG effect, respectively. The second-order nonlinearity mode at wavelengths 451 nm is the sum frequency field signals for two incident continuous waves with wavelengths 636 nm and 1548 nm. And the SH conversion efficiencies of sum-frequency field signal for two fundamental frequency wave incidence is about 10^−13^ or 10^−14^. The second-order nonlinearity mode at wavelength 1080 nm is the difference frequency field for two incident continuous waves with wavelengths 636 nm and 1548 nm. The transmission of the fundamental light results from an enhancement of the local field. The strong local SP resonance induces an increase of the four second-order nonlinearity signals. The enhancement of the second-order nonlinearity signals include second-harmonic generation as well as the sum (difference) frequency generation.

## 4. Conclusions

Unlike SGH in square lattice perforated gold film in many papers, we have studied the excitation of second-order nonlinearity signals through a triangular lattice perforated gold film due to surface plasmas resonance effect. Based on the FDTD method, the SH conversion efficiencies in the second-order nonlinear optical process are studied. The electric field distributions of fundamental frequency are calculated. The second-order nonlinearity phenomenon including second-harmonic generation as well as the sum (difference) frequency generation is shown in our paper.
